# Prospective comparison of ^68^Ga-DOTA-ibandronate and bone scans for detecting bone metastases in breast cancer

**DOI:** 10.3389/fonc.2024.1428498

**Published:** 2024-07-31

**Authors:** Feifan Xiang, Yue Zhang, Xiaoqi Tan, Yuanzhuo Yan, Huipan Liu, Wenzhe Ma, Yue Chen

**Affiliations:** ^1^ The State Key Laboratory of Quality Research in Chinese Medicine, Macau University of Science and Technology, Macao, Macao SAR, China; ^2^ Department of Orthopedic, The Affiliated Hospital, Southwest Medical University, Luzhou, China; ^3^ Department of Nuclear Medicine, The Affiliated Hospital, Southwest Medical University, Luzhou, China; ^4^ Department of Dermatology, The Affiliated Hospital, Southwest Medical University, Luzhou, China; ^5^ Institute of Nuclear Medicine, Southwest Medical University, Luzhou, China; ^6^ Nuclear Medicine and Molecular Imaging Key Laboratory of Sichuan Province, Luzhou, China

**Keywords:** breast cancer, positron emission tomography, whole-body bone scan, bone metastases, computed tomography

## Abstract

**Introduction:**

^68^Ga labeled DOTA-Ibandronate (^68^Ga-DOTA-IBA) positron emission tomography/computed tomography (PET/CT), is a novel bone-targeting imaging tracer and promising diagnostic method for bone metastases detection. Therefore, this study aimed to compare ^68^Ga-DOTA-IBA PET/CT to the ^99m^Tc-MDP whole-body bone scan (WBBS) for detecting bone metastases in breast cancer (BC).

**Materials and methods:**

In this prospective study, 45 women with BC underwent imaging via ^68^Ga-DOTA-IBA PET/CT and ^99m^Tc-MDP WBBS. Clinical and demographic information as well as BC imaging features were recorded. The two methods were compared in terms of their detection rate for bone metastases and the number of lesions.

**Results:**

The 45 women were aged 53.5 ± 11.0 years. The bone metastases detection rate with ^68^Ga-DOTA-IBA PET/CT was 100% (45/45) and with ^99m^Tc-MDP WBBS was 95.6% (43/45). A total of 546 bone metastases lesions were detected. The lesion detection rate using ^68^Ga-DOTA-IBA PET/CT was 100% (546/546) and using ^99m^Tc-MDP WBBS was 67.8% (370/546). More lesions were found at each site via ^68^Ga-DOTA-IBA than via ^99m^Tc-MDP WBBS.

**Conclusions:**

^68^Ga-DOTA-IBA PET/CT is a more sensitive method than ^99m^Tc-MDP WBBS for assessing bone metastases in BC and may therefore represent a useful imaging technique for bone metastases, while offering a visual basis for ^177^Lu-DOTA-IBA diagnosis and therapy response assessments for BC. Further validation using a broader study cohort is warranted to confirm these findings.

**Clinical trial registration:**

https://www.chictr.org.cn/showproj.html?proj=170163, identifier ChiCTR2200064487.

## 1 Introduction

Breast cancer (BC) is the primary cause of cancer-related illness, impairment, and mortality in women worldwide (1). Bones are the most common sites of distant metastases in BC and impact prognosis, quality of life, and therapy, which may affect approximately 65–90% of patients with advanced illnesses (2). Patients with less metastatic diseases have better prognoses, and those who have primarily bone-related metastases have higher survival rates than those with visceral metastases—suggesting that prognosis may be influenced by early detection. Therefore, early diagnosis and therapy response monitoring are essential in these patients (3).

Conventional imaging techniques such as radiography, computed tomography (CT), and magnetic resonance imaging (MRI) are considered to be inadequate for detecting and accurately assessing the effectiveness of BC treatment. While the ^99m^Tc-MDP whole-body bone scan (WBBS) is commonly recommended, its sensitivity and specificity are not ideal (4, 5). Therefore, it is advisable to perform ^18^F-fludeoxyglucose (FDG) positron emission tomography (PET)/CT after conducting CT and WBBS of the chest, abdomen, and pelvis for stage IIA–IIIC BC (6). Compared with other methods, single-photon emission computed tomography (SPECT)/CT and PET/CT exhibit greater accuracy for bone staging diagnosis and provide the possibility for early individualized treatment. However, SPECT/CT may lead to false positive diagnoses or missed diagnoses. Moreover, the ^18^F-FDG PET/CT method has a limited ability to detect skull metastases and lacks specificity in identifying bone lesions (7). Nevertheless, treatments for BC bone metastases have become possible with the development of integrated probes for molecular-targeted diagnosis and treatment in nuclear medicine. Thus, accurate detection and monitoring of the response to bone metastases has gained importance (8).

The ^68^Ga or ^177^Lu labeled DOTA-Ibandronate (^68^Ga/^177^Lu-DOTA-IBA) approach, an integrated probe for both diagnosis and treatment exhibits strong targeting of hydroxyapatite, low uptake in background organs, and long retention time in bone metastases (9, 10). Preliminary clinical studies have shown that ^68^Ga-DOTA-IBA PET/CT detects more bone metastases in various solid tumors compared with WBBS or Sodium fluoride PET/CT (^18^F-NaF). However, the previous studies only evaluated several cases of BC (11, 12). ^177^Lu-DOTA-IBA treated bone metastases without significant liver and kidney function damage and bone marrow suppression; patient symptoms significantly improved and the short-term curative effect was definite (11). Thus, the accurate detection of more bone metastases by ^68^Ga-DOTA-IBA may also contribute to the early treatment of ^177^Lu-DOTA-IBA. Therefore, this study aimed to compare ^68^Ga-DOTA-IBA PET/CT with WBBS for detecting BC bone metastases and provide evidence for the use of ^177^Lu-DOTA-IBA as a BC treatment.

## 2 Materials and methods

### 2.1 Participants

The Affiliated Hospital of Southwest Medical University served as the site of this prospective, single-center study. Participants were consecutively enrolled between January 2022 and October 2023 (clinical trial registration no. ChiCTR2200064487; Ethics Committee Approval No. KY2022114). All participants provided written informed consent before undergoing ^68^Ga-DOTA-IBA PET/CT imaging. Following registration, the individuals underwent a PET/CT scan using ^68^Ga-DOTA-IBA and a WBBS within one week. All participants were followed-up for a minimum period of 3 months.

Eligible participants were required to meet the following criteria: (a) recently diagnosed, recurring, or spreading BC; (b) BC confirmed through histological examination; and (c) willingness to undergo ^68^Ga-DOTA-IBA PET/CT and WBBS scans. Patients who had additional primary malignancies during the examination, severe hepatic, or renal insufficiency, or declined to undergo ^68^Ga-DOTA-IBA PET/CT were excluded. Biopsies and histopathological examinations were used to diagnose both initial and recurring cases of BC. Diagnoses of bone metastases were established using multiple imaging modalities, including brain MRI, chest and abdominal CT, WBBS, and PET/CT. Owing to the advanced stage of the participants’ illnesses, only a limited number of biopsies were performed to investigate potential metastatic lesions.

### 2.2 ^68^Ga-DOTA-IBA PET/CT and WBBS imaging

Imaging was performed according to a previously described protocol (11). No specific preparations were required before the examinations and 1.85 MBq (0.05 mCi) per kilogram body weight of ^68^Ga-DOTA-IBA was administered through intravenous injection. A PET/CT scan was performed 40–60 min after the tracer was injected, covering the entire body from head to toe, with 3 min per position. The resulting images underwent attenuation correction and iterative reconstruction to obtain transverse, coronal, and sagittal PET/CT scan views. The WBBS was performed 3–4 h following the intravenous administration of 740–925 MBq (20–25 mCi) of ^99m^Tc-MDP.

### 2.3 Imaging analysis

Two trained and board-certified nuclear medicine doctors independently analyzed the obtained images to compare the detection of bone metastases between the two methods. Any disagreements were resolved through discussion. Individual skeletal metastases were classified into nine regions: the cervical spine, thoracic spine, lumbosacral spine, pelvis, long bone and clavicle, craniofacial bone, scapula, rib, and sternum. We recorded the number of osteoarticular lesions with abnormal tracer uptakes on PET/CT or WBBS, along with their sites, any abnormal CT findings, and the maximum standardized uptake value (SUVmax). Metastases to the same vertebral body or appendage were identified as a single lesion. The imaging features of PET/CT and bone scans were analyzed, and the detection rates of the bone metastases and the number of lesions using the two methods were calculated.

### 2.4 Statistical analysis

SPSS Statistics version 26.0 (IBM) was used for the data analysis. Descriptive statistics are shown as either means ± standard deviations, medians (ranges), or numbers (%). The paired Chi-square test (McNemar test) was used to compare the detection rates of ^68^Ga-DOTA-IBA PET/CT and WBBS. P < 0.05 was considered statistically significant.

## 3 Results

### 3.1 Participant cohort

Between January 2022 and October 2023, 45 patients diagnosed with BC, with an average age of 53.5 ± 11.0 years, were included in this study. Histopathological analyses revealed six cases of triple-negative BC. Surgery, chemotherapy, radiotherapy, targeted therapy, and endocrine therapy were performed at least 3 months before each examination (**Table 1**).

**Table 1:** Demographic and clinical features of the 45 participants with breast cancer.

Demographic and clinical features (N = 45)	
Age, mean ± SD, y	53.5 ± 11.0
Site, n (%)	
left	21 (46.7)
right	24 (53.5)
WHO, n (%)	
WHO II	24 (53.5)
WHO III	21 (46.7)
Histopathologic findings, n (%)	
NTNBC	39 (86.7)
TNBC	6 (13.3)
Treatment, n (%)	
surgery	38 (84.4)
radiotherapy	12 (26.7)
chemotherapy	34 (75.6)
targeted therapy	10 (22.2)
endocrine therapy	10 (22.2)

WHO, World Health Organization; NTNBC, non-triple negative breast cancer; TNBC, triple negative breast cancer.

### 3.2 Imaging characteristics

Bone metastases were detected in all 45 patients. WBBS did not detect bone metastasis in two patients, resulting in a detection rate of 95.6% (43/45). Whereas ^68^Ga-DOTA-IBA PET/CT detected bone metastases in all patients, exhibiting a detection rate of 100% (45/45) (P > 0.05). The number of patients detected as having scapular lesions was similar across the two methods (16/16), whereas ^68^Ga-DOTA-IBA PET/CT detected more patients with other lesions than WBBS. A significantly higher number of patients were detected with lesions in the cervical (24/16) and thoracic vertebrae (25/20) using ^68^Ga-DOTA-IBA PET/CT than using WBBS (**Table 2**). The diagnosis of bone metastasis was confirmed by pathological examination in only five patients (five lesions) who visited the orthopedic department due to bone-related events.

**Table 2:** Bone-positive Lesions Detected by ^68^Ga-DOTA-IBA PET/CT and ^99m^Tc-MDP Bone Scan.

Site	^99m^ Tc- MDP Bone Scan		^68^Ga-DOTA-IBA PET/CT		
	Patients	Lesions	Patients	Lesions	SUVmax Median (Range)
Cervical spine	16	20	24	44	5.39 (4.29–6.99)
Thoracic spine	20	60	25	107	6.91 (5.17–10.78)
Lumbosacral spine	30	77	31	92	8.66 (5.85–13.15)
Pelvis	18	53	21	79	7.27 (4.67–11.14)
Long bone and clavicle	15	41	16	52	6.81 (4.85–10.23)
Craniofacial bone	12	15	13	18	7.01 (4.62–11.06)
Scapula	16	21	16	25	5.59 (4.38–11.23)
Rib	24	64	26	107	5.18 (3.99–8.40)
Sternum	18	19	21	22	5.70 (3.87–12.72)
Total	–	370	–	546	6.57 (4.76–10.30)

IBA, ibandronate.

“-” indicates that the number of patients is not summarized.

A total of 546 bone metastases were detected in 45 patients, 370 of which were detected using WBBS. The lesion detection rate was 67.8% (370/546) via WBBS and 100% (546/546) via ^68^Ga-DOTA-IBA (P < 0.001).

**Figure 1:**
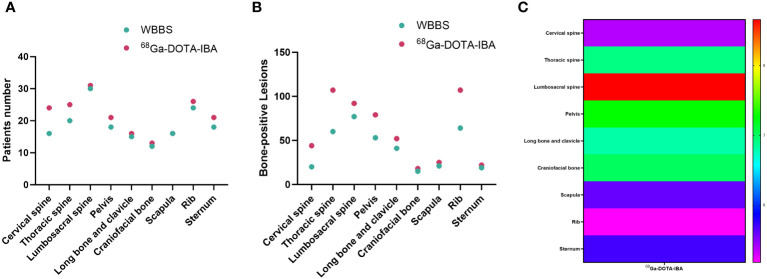
Comparison of ^68^Ga-DOTA-ibandronate (IBA) PET/CT and ^99m^Tc-MDP bone scan (WBBS) in detecting bone metastases in breast cancer. **(A)** Number of patients testing positive; **(B)** Number of positive lesions; **(C)** Heat maps showing the SUVmax of ^68^Ga-DOTA-IBA in bone metastases at various anatomical sites.

**Figure 2:**
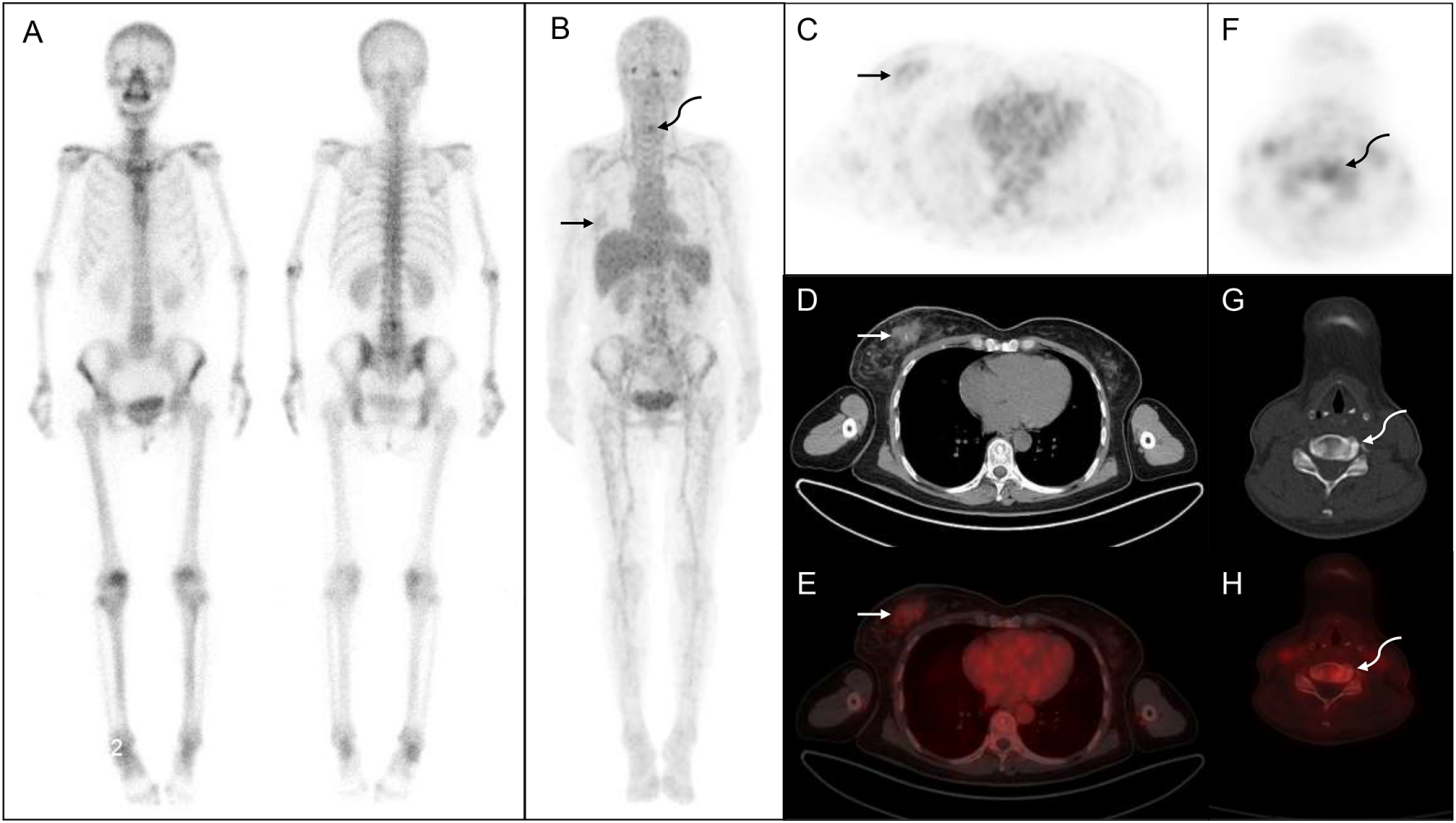
^68^Ga-DOTA- ibandronate (IBA) PET/CT performed on a 54-year-old woman with breast cancer. A ^99m^Tc-MDP bone scan **(A)** shows no significant abnormalities, whereas a ^68^Ga-DOTA-IBA PET/CT **(B)** shows increased tracer uptake in the right mammary gland (straight arrow) and cervical spine (curved arrow). **(C–E)** shows right breast nodules with abnormal tracer uptake (SUVmax, 1.84; straight arrow). **(F–H)** shows a cervical bone change with increased metabolism (SUVmax, 3.46, curved arrow) that is subsequently confirmed as invasive cancer via biopsy.

The lesion detection rate of ^68^Ga-DOTA-IBA in the central bone was significantly higher than that of WBBS. For all lesions, the median SUVmax of ^68^Ga-DOTA-IBA was 6.57 (range: 4.76–10.30; **Table 2**, **Figure 1**). Notably, ^68^Ga-DOTA-IBA PET/CT showed increased uptake of DOTA-IBA at the primary BC site in two patients (**Figure 2**). The ^68^Ga-DOTA-IBA PET/CT imaging results of one out of the two patients were previously reported (13).

## 4 Discussion

This study prospectively evaluated the diagnostic accuracy of ^68^Ga-DOTA-IBA PET/CT and WBBS for detecting bone metastases in BC. The bone metastases were divided into nine groups for individual assessment. The overall detection rates between ^68^Ga-DOTA-IBA PET/CT and WBBS were not significantly different, at 100% and 95.6%, respectively, P > 0.05. However, in terms of the lesion detection rate, ^68^Ga-DOTA-IBA PET/CT was superior to WBBS (100% vs. 67.8%, respectively, P < 0.05). Overall, ^68^Ga-DOTA-IBA PET/CT detected more bone metastases than WBBS.

**Figure 3:**
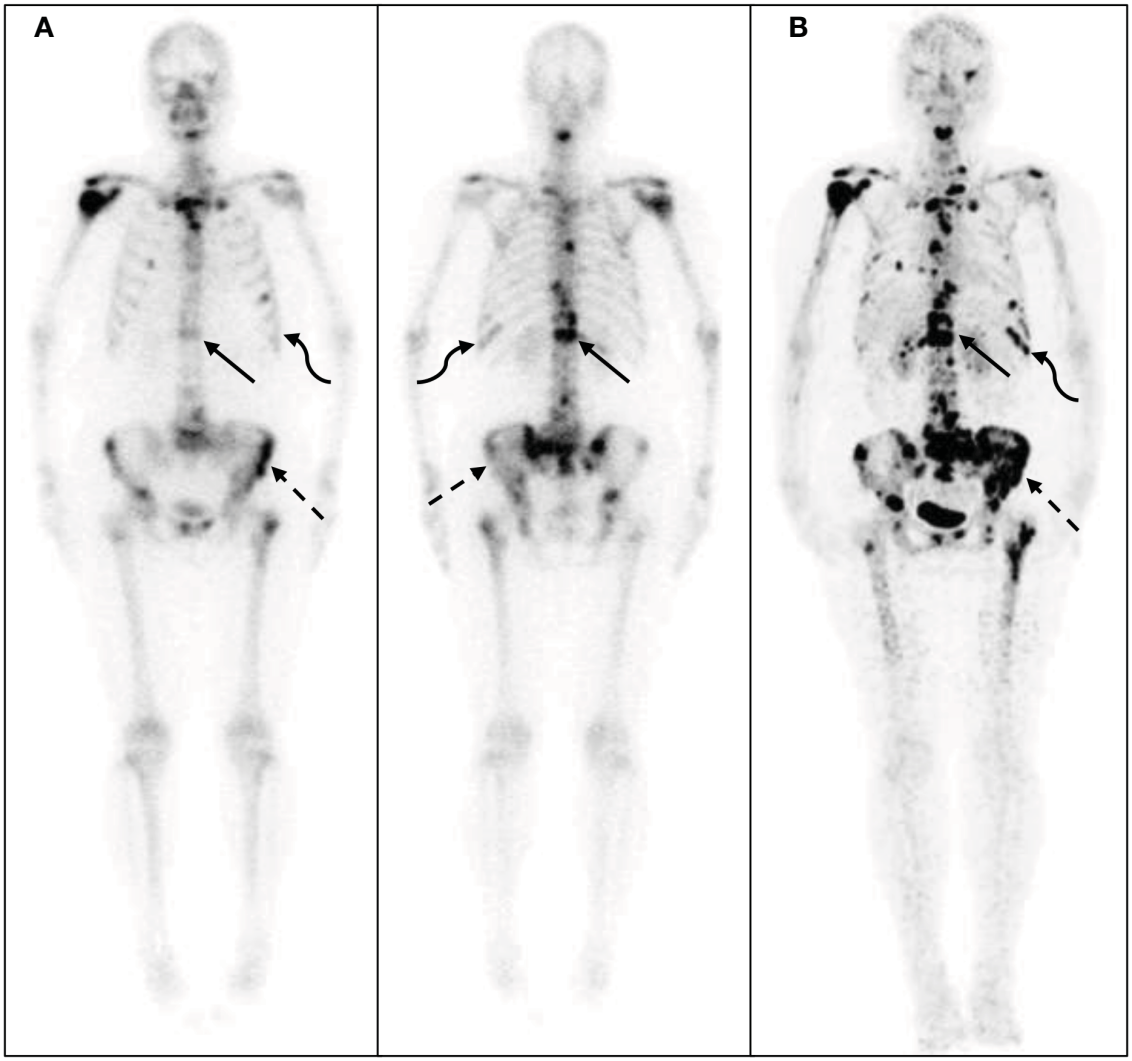
^68^Ga-DOTA- ibandronate (IBA) PET/CT performed on a 58-year-old woman with breast cancer 10 years following surgical treatment. A ^99m^Tc-MDP bone scan **(A)** shows no significant increase in bone metabolism, whereas a ^68^Ga-DOTA-IBA PET/CT shows **(B)** multiple bone metastases throughout the body and significantly increased bone metabolism in the ribs (straight arrow), spine (dotted arrow), and sternum (curved arrow).

**Figure 4:**
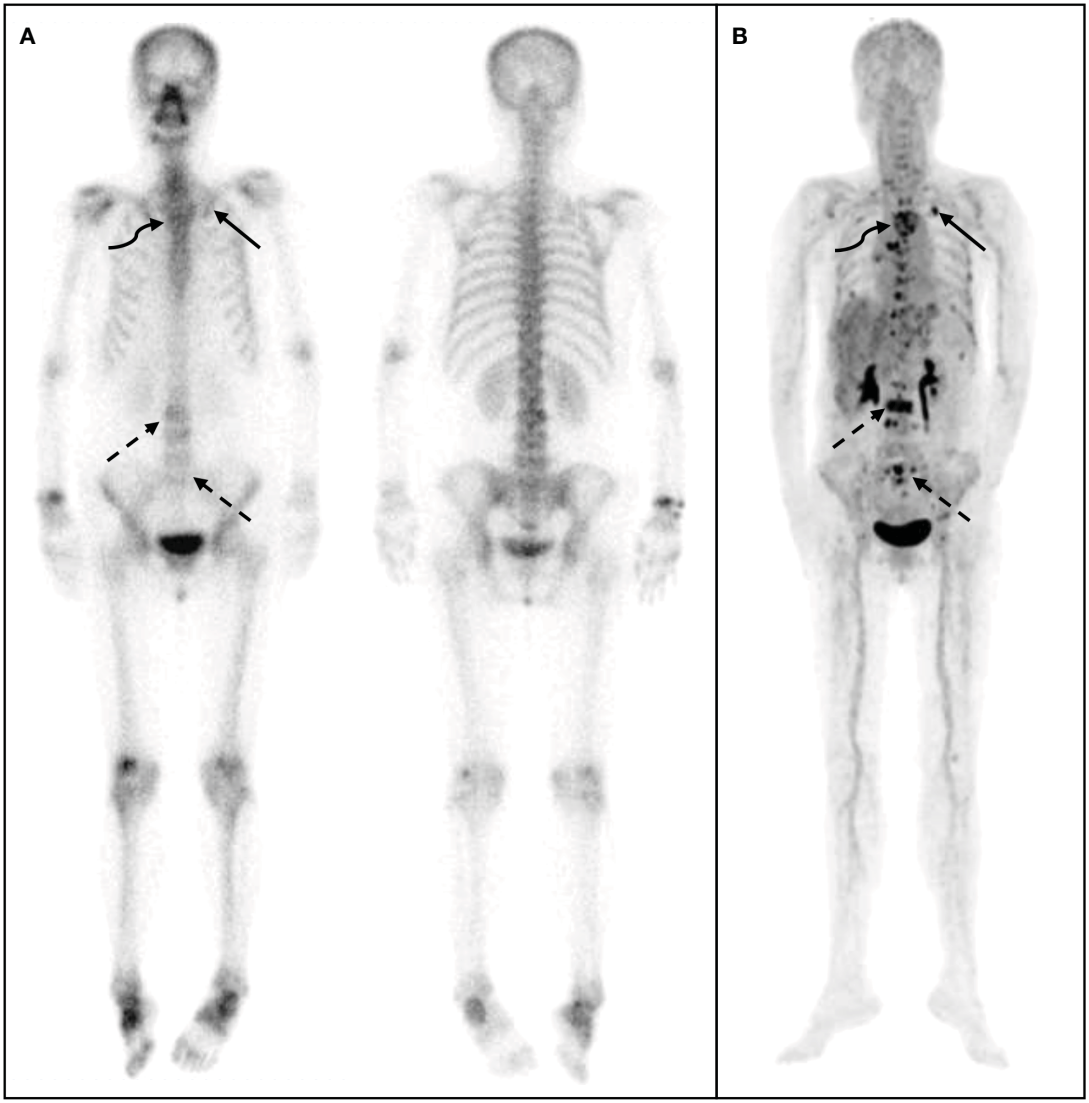
^68^Ga-DOTA- ibandronate (IBA) PET/CT performed on a 48-year-old woman with invasive cancer 4 years following surgical treatment for BC. A ^99m^Tc-MDP bone scan **(A)** shows multiple bone metastases in sites such as the vertebral body, ribs, ilium, and humerus. A ^68^Ga-DOTA-IBA PET/CT **(B)** reveals more vertebral (straight arrow) and rib (curved arrow) metastases, particularly in the pelvis (dotted arrow).

The ^68^Ga-DOTA-IBA PET/CT approach revealed multiple significant bone metastases in the vertebral body in cases where WBBS only showed suspicious metastases in the area; this could potentially affect the clinical treatment plans (**Figure 3**). Moreover, when bone metastases were detected by WBBS, ^68^Ga-DOTA-IBA accurately displayed multiple bone metastases with high SUVmax uptake levels, which is beneficial for radionuclide-targeted therapy and post-treatment evaluation (**Figure 4**). In recent years, ^18^F-FDG PET/CT has become a valuable technique for staging BC (14–16). However, there is ongoing debate regarding the accuracy and sensitivity of PET/CT for identifying bone metastases when compared with WBBS (17). Some scholars believe that its accuracy and sensitivity for diagnosing skull metastases are higher than those of PET/CT (18). In this study, ^68^Ga-DOTA-IBA PET/CT was superior to bone scans for the diagnosis of skull metastases due to the type of molecular probe used. The high uptake of FDG in brain tissues may mask skull metastases and interfere with diagnoses (19, 20), whereas ^68^Ga-DOTA-IBA avoids this shortcoming (**Figure 4**). Additionally, there is a higher uptake of ^68^Ga-DOTA-IBA PET/CT in non-calcified BC tissues, which may be caused by localized increase in blood pool, calcium metabolism, or interstitial volume in the breast tissue (21).

Thus, compared with WBBS, ^68^Ga-DOTA-IBA PET/CT can be used to obtain an earlier diagnosis of bone metastases in BC. The ^68^Ga-DOTA-IBA targets hydroxyapatite, which has a higher uptake at bone metastasis sites and is more sensitive to osteolysis, making it beneficial for the early diagnosis of bone metastases (9). In contrast, PET/CT has the advantages of providing full imaging and anatomical localization that can be used to accurately locate the sites of bone metastases (22, 23). However, WBBS, which lacks anatomical localization, may lead to errors in diagnosis (24). ^18^F-FDG PET/CT is routinely used for systemic staging, monitoring treatment response and recurrence of advanced BC (stage IIB-IIIC). A previous study compared the ^18^F-FDG alternative with WBBS for evaluating bone metastases in patients with recently detected metastatic BC; WBBS provided insufficient information and warranted an additional evaluation via ^18^F-FDG PET/CT in > 25% of the patients (25). However, ^18^F-FDG does not target the bone, and the sensitivity and accuracy of its diagnoses vary because of the tumor heterogeneity (26). Additionally, ^18^F-NaF PET/CT is more sensitive to bone metastases than ^99m^Tc-MDP or CT in patients with metastatic BC (27). A prospective comparison showed that ^18^F-NaF PET/CT was more accurate than SPECT for the diagnosis of BC bone metastases (28). Consistent with previous findings, PET/CT showed advantages over WBBS for the diagnosis of BC bone metastases in this study. However, different molecular probes have different diagnostic accuracies and sensitivities for PET/CT, and most can only diagnose bone metastases, lacking diagnosis and treatment integration (29, 30). Therefore, the diagnostic efficacy of ^68^Ga-DOTA-IBA PET/CT compared with ^18^F-NaF PET/CT or ^18^F-FDG PET/CT, which are more sensitive to detect BC bone metastases, requires further investigation.

The previously developed ^68^Ga/^177^Lu-DOTA-IBA approach is an integrated probe for both diagnosis and treatment. The SUVmax of ^68^Ga-DOTA-IBA for detecting bone metastases was found to be higher than that of WBBS in our patient cohort; the whole-body SUVmax of bone metastases was 6.57, and the highest value was found in the lumbosacral vertebrae (8.66; **Table 2**). Thus, ^68^Ga-DOTA-IBA can be used to diagnose and evaluate bone metastases in patients with BC who have bone metastases. Additionally, ^177^Lu-DOTA-IBA has a high therapeutic effect that can rapidly relieve bone pain due to cancerous lesions (11, 31). We found that ^68^Ga-DOTA-IBA tracer uptake was also present in the primary lesion of two patients, suggesting that ^177^Lu-DOTA-IBA may have a therapeutic effect on the primary lesion in addition to targeting the bone metastases. While some radiopharmaceuticals like ^89^Sr, ^223^Ra, ^188^Re/^186^Re-HEDP, ^153^Sm-EDMTP, ^177^Lu-EDTMP, etc, are currently used for treating bone metastases and have demonstrated high analgesic potential, they are not suitable for therapeutic use due to the absence of corresponding diagnostic analogs (11). Nevertheless, this integrated diagnosis and treatment probe (^68^Ga/^177^Lu-DOTA-IBA) has potentially broad applications for the diagnosis and treatment of bone metastases resulting from solid tumors.

Despite the promising results, this study had some limitations worth noting. First, the number of enrolled patients was small, and their cancer stages were not discussed. Due to the limited number of different stages of the patients included in this study, whether ^68^Ga-DOTA-IBA can impact patient management requires further evaluation with a larger sample cohort of patients at different stages. Second, most of the patients did not have pathological examination results, which were mainly evaluated using imaging and comprehensive treatment responses. Third, ^68^Ga-DOTA-IBA PET/CT was not compared to ^18^F-NaF PET/CT or ^18^F-FDG PET/CT. Finally, the follow-up period was relatively short. Therefore, future multi-center and longer-term studies addressing these limitations are warranted to validate our results.

## 5 Conclusion

^68^Ga-DOTA-IBA PET/CT, compared to the WBBS, represents a more precise method for the detection of bone metastases in BC. It offers a potential imaging method and establishes a basis for diagnoses and treatment response evaluations using ^177^Lu-DOTA-IBA. However, further validation using a broader study cohort is warranted.

## Data availability statement

The original contributions presented in the study are included in the article/supplementary material. Further inquiries can be directed to the corresponding authors.

## Ethics statement

The studies involving humans were approved by Affiliated Hospital of Southwest Medical University. The studies were conducted in accordance with the local legislation and institutional requirements. The participants provided their written informed consent to participate in this study. Written informed consent was obtained from the individual(s) for the publication of any potentially identifiable images or data included in this article.

## Author contributions

FX: Writing – review & editing, Writing – original draft, Data curation, Conceptualization. YZ: Methodology, Writing – review & editing, Writing – original draft, Investigation. XT: Writing – review & editing, Formal analysis, Data curation. YY: Writing – review & editing, Software, Formal analysis. HL: Validation, Data curation, Writing – review & editing. WM: Writing – review & editing, Validation, Supervision, Project administration. YC: Writing – review & editing, Resources, Project administration, Funding acquisition.
